# Frontal Sinus Balloon Sinuplasty—Patient Satisfaction and Factors Predicting Reoperation

**DOI:** 10.1002/oto2.23

**Published:** 2023-03-22

**Authors:** Sara Sainio, Karin Blomgren, Anni Koskinen, Marie Lundberg

**Affiliations:** ^1^ Department of Otorhinolaryngology‐Head and Neck Surgery Helsinki University Hospital and University of Helsinki Helsinki Finland; ^2^ HUS Joint Resources Helsinki University Hospital and University of Helsinki Helsinki Finland; ^3^ Department of Clinical Allergy and Immunology, Brigham and Women's Hospital Harvard Medical School Boston Massachusetts USA

**Keywords:** balloon dilation, balloon sinuplasty, frontal sinuplasty, frontal sinus ostium, long‐term outcome

## Abstract

**Objective:**

To explore predictive factors of postoperative outcome of frontal sinus balloon dilation.

**Study Design:**

Retrospective questionnaire study.

**Setting:**

Department of Otorhinolaryngology–Head and Neck Surgery, Helsinki University Hospital and University of Helsinki, Finland.

**Methods:**

We reviewed electronic records of all patients who underwent frontal sinus balloon dilatation (successful or attempted) in our clinic from 2008 to 2019. We documented patient characteristics, preoperative imaging results, intraoperative factors, possible complications, and reoperations. Those who underwent frontal sinus balloon sinuplasty were sent a questionnaire regarding their current symptoms and long‐term satisfaction with surgery.

**Results:**

In total, 258 operations (404 frontal sinuses) were reviewed, with a technical success rate of 93.6% (n = 378). The revision rate was 15.7% (n = 38). Previous sinonasal surgery predicted a higher revision rate (*p* = .004, odds ratio [OR] = 3.03, 95% confidence interval [CI] 1.40‐6.56). Patients with hybrid surgery had significantly fewer reoperations compared to the balloon only group (*p* = .002, OR = 0.33, 95% CI 0.16‐0.67). The response rate of the questionnaire was 64.5% (n = 156), of which 88.5% (n = 138) reported a long‐term benefit from the balloon sinuplasty. Patient satisfaction was higher (*p* = .02, OR = 8.26, 95% CI 1.06‐64.24) among patients using nasal corticosteroids.

**Conclusion:**

Technical success rate and patient satisfaction after frontal sinus balloon sinuplasty are high. Balloon sinuplasty seems insufficient in reoperations. A hybrid approach appears to result in fewer reoperations than a balloon only approach.

Balloon dilation of sinus ostia was introduced in 2005 as a novel surgical method for treating recurrent acute rhinosinusitis and chronic rhinosinusitis.^1^, ^2^

Balloon sinuplasty is considered a safe and effective treatment for selected cases of recurrent acute and chronic rhinosinusitis of frontal, sphenoid, and maxillary sinuses.^1^, ^2^, ^3^, ^4^, ^5^, ^6^, ^7^, ^8^, ^9^ It is less invasive than conventional endoscopic sinus surgery (ESS), patient recovery is faster, and due to lesser mucosal trauma, it seems to entail less risk for postoperative scarring, especially in the frontal recess.^1^, ^3^, ^10^ Balloon sinuplasty is not considered an effective treatment for chronic rhinosinusitis with nasal polyposis, aspirin‐exacerbated respiratory disease, or allergic fungal rhinosinusitis.^1^, ^11^

Using balloon sinuplasty is especially attractive in the frontal sinus because of the demanding conventional surgery.^2^, ^10^, ^12^, ^13^, ^14^, ^15^ Possible complications of sinuplasty relate to the proximity of the skull base, orbit, and anterior ethmoidal artery.^13^ Although major complications are rare, cases of cerebrospinal fluid leaks, orbital injuries, and major bleeding have been reported.^16^, ^17^, ^18^, ^19^, ^20^

Long‐term results of balloon dilation of the frontal sinus ostium, factors predicting success, or the need for reoperations are not fully known. This study aimed to address these questions to optimally identify patients who reap long‐lasting benefits from balloon sinuplasty.

## Methods

We reviewed the electronic patient database of the Department of Otorhinolaryngology–Head and Neck Surgery, Helsinki University Hospital (HUS). We identified patients operated on between 2008 and 2019 with the surgical codes DPA20 (external frontal sinusotomy), DPA25 (endoscopic frontal sinusotomy), and ZXB87 (sinonasal balloon dilation) according to the Nordic Classification of Surgical Procedures (NCSP). We documented technically successful balloon sinuplasties as one group and failed attempts as another. A technically successful operation was defined as the surgeon being able to insert the balloon into the frontal sinus ostium and perform the dilation, and unsuccessful as cases in which the surgeon deemed it necessary to switch to conventional equipment as the balloon could not be inserted properly. The reasons for technical failure were reviewed.

From patient records, we documented age, sex, smoking status, allergies, bronchial asthma, comorbidities, preoperative peroral use of corticosteroids, previous sinonasal surgeries, and the indication for surgery marked in the patient records (pain, pressure‐adjusting problems, enlarging a previously dilated frontal canal, or infections, that is an acute bacterial frontal sinus disease not resolving with conservative treatment). Elixhauser Comorbidity Index describing the risk of postoperative complications was calculated for each patient based on the patients' comorbidities, with the score ranging from −19 (minimal risk for death in hospital) to 89 (maximum risk for death in hospital).^21^ All patients' preoperative computed tomography (CT) scans were reviewed and scored with Lund‐MacKay score for all paranasal sinuses and Zinreich score for the frontal sinuses, that is, each frontal sinus was graded 0 to 5 points based on its opacification (0 = 0%, 1 = 1%‐25%, 2 = 26%‐50%, 3 = 51%‐75%, 4 = 76%‐99%, 5 = 100%).^22^, ^23^ The status of the frontal recess was noted (open or closed).

From surgical records, we reviewed the type of anesthesia, American Society of Anesthesiologists (ASA) classification, manufacturer of the balloon catheter (Acclarent [Irvine], Entellus [Plymouth], or other), other simultaneous sinonasal surgeries in conjunction with balloon sinuplasty (conventional surgery of maxillary or sphenoidal sinuses, ethmoidectomy, polypectomy, septoplasty, minitrephination), frontal sinus findings, possible perioperative complications, and use of packing.

We reviewed complications that occurred within 30 days of surgery, including infection, bleeding requiring treatment, orbital fractures, skull base fractures, and intracranial complications. We also recorded possible reoperations occurring at any time point between initial surgery and end of study in 2020, and noted both indication and the time interval between balloon sinuplasty and the decision for reoperation.

We developed a questionnaire (Table 1) to confirm long‐term results, which EUPATI Finland (European Patients Academy on Therapeutic Innovation) reviewed to ensure the questions were clear to the patients. All patients with technically successful frontal sinus balloon sinuplasty were asked to participate in 2020, first electronically and, if no answer was received, by mail. In conjunction with the questionnaire, patients also answered the Sino‐Nasal Outcome Test‐22 (SNOT‐22).

**Table 1:** Patient Questionnaire and Response Rates for Survey Sent to Patients Who Underwent Frontal Sinus Balloon Sinuplasty at Helsinki University Hospital From 2008 to 2019

Question	Answer	N (%)
Have you used antibiotics for sinusitis during the last 12 months?	Yes	59 (37.8)
	No	97 (62.2)
Have you used nasal corticosteroids during the last 12 months?	Yes	107 (68.6)
	No	48 (30.8)
Were you recommended to use nasal corticosteroids regularly after the balloon sinuplasty?	Yes	78 (50.0)
	No	30 (19.2)
	Unknown	48 (30.8)
Do you regularly use any other local nasal treatment(s)?	Yes	75 (48.1)
	No	81 (51.9)
If yes to the above, what other local treatment(s)?	Moisturizing nasal spray	29 (18.6)
	Saline irrigation	58 (37.2)
	Nasal decongestant	10 (6.4)
Do you smoke daily?	Yes	12 (7.7)
	No	117 (75.0)
	Ex‐smoker	27 (17.3)
If you are a smoker, how many cigarettes do you smoke per day? Median (IQR).		4 (6)
Have you had any sinonasal surgeries performed outside Helsinki University Hospital after your balloon sinuplasty?	Yes	3 (1.9)
	No	153 (98.1)
Do you suffer from problems adjusting sinus pressure?	Yes	63 (40.4)
	No	93 (59.6)
Do you suffer from pain in the forehead during infections?	Yes	104 (66.7)
	No	52 (33.3)
Do you suffer from pain in the forehead without any specific reason?	Yes	58 (37.2)
	No	98 (62.8)
Do you think you benefitted from the operation?	Yes	138 (88.5)
	No	18 (11.5)
Estimate how your symptoms changed after the operation.	I became symptom‐free	46 (29.5)
	The symptoms decreased	92 (59.0)
	The symptoms remained the same	16 (10.3)
	The symptoms increased a little	2 (1.3)
	The symptoms increased markedly	0
SNOT‐22, total score, median (IQR)		22 (25)

Abbreviations: IQR, interquartile range; SNOT‐22, Sino‐Nasal Outcome Test‐22.

The data were analyzed using NCSS 12 Statistical Software (2018, NCSS, LLC, ncss.com/software/ncss), applying the functions cross‐tabulation, 2‐sample *t*‐test, and analysis of variance (ANOVA) in normally distributed values, and the Mann‐Whitney *U* test and Kruskal‐Wallis test in values not normally distributed. Odds ratios were analyzed by applying logistic regression analysis. A *p* < .05 was considered statistically significant.

The Helsinki University Ethics Research Board (HUS/3401/2019) approved the study protocol. All patients who returned the questionnaire gave their informed written consent.

## Results

### Study Population and Technical Success Rate

**Figure 1:**
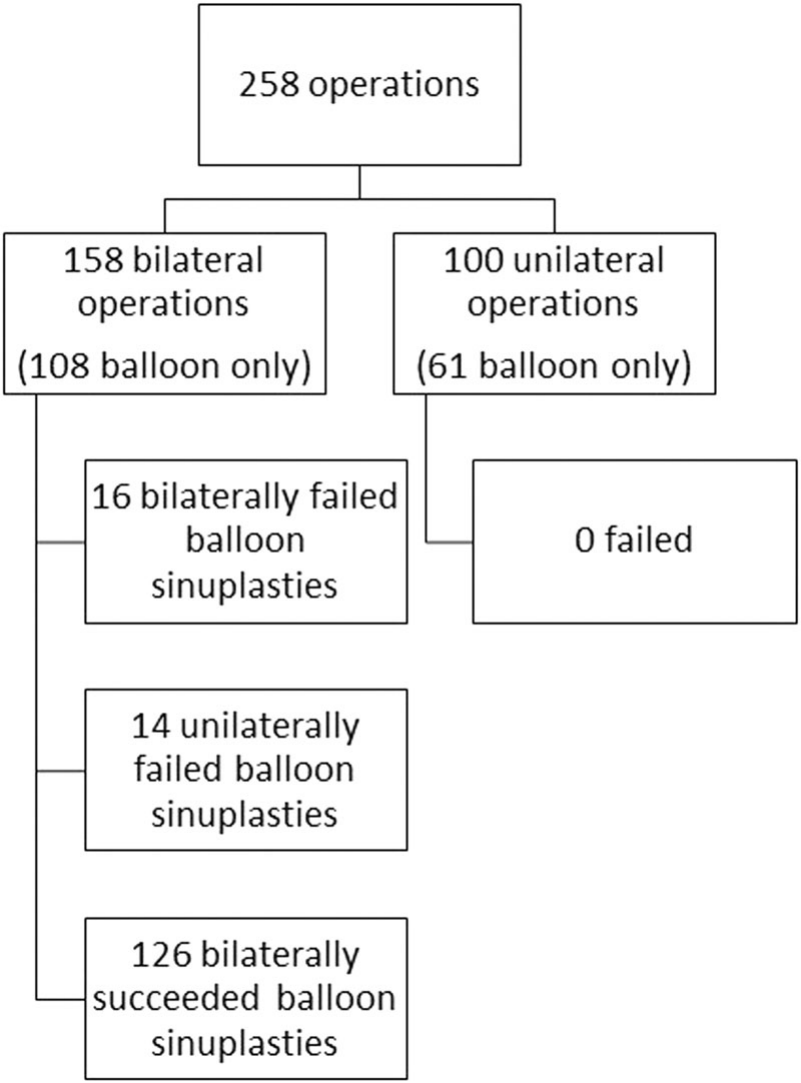
Flow chart of attempted frontal sinus balloon sinuplasties performed at Helsinki University Hospital from 2008 to 2019.

Our data included 259 technically successful or attempted frontal balloon sinuplasties at HUS 2008 to 2019, with a median follow‐up time (interquartile range [IQR]) of 54 months (35 months). One patient withdrew their informed consent and was excluded, leaving 258 operations (Figure 1). Of the 404 attempted frontal sinus ostia, 378 (93.6%) were technically successfully dilated with balloon sinuplasty by 19 surgeons. Cases, where the surgeon had to switch to conventional ESS, were considered failures. Two hundred and fourteen (82.9%) operations were performed under general anesthesia, 44 (17.1%) under local anesthesia in operating room setting. Seven patients underwent frontal balloon sinuplasty twice or more. In these cases, we reviewed each surgery individually, and the patients were asked to answer the questionnaire regarding the latest operation. Patient characteristics for the technically successful and unsuccessful balloon sinuplasty groups are in Table 2.

**Table 2:** Characteristics of Patients Who Underwent Technically Successful Versus Technically Failed Frontal Sinus Balloon Sinuplasty at Helsinki University Hospital From 2008 to 2019

	Technically successful group	Technically failed group	OR (95% CI)
Sex, n (%)			
Women	114 (50.0)	14 (46.7)	1
Men	114 (50.0)	16 (53.3)	1.14 (0.53‐2.45)
Age, y, mean (95% CI)	44.7 (42.8‐46.6)	48.0 (43.0‐53.0)	1.02 (0.99‐1.04)
BMI, median (IQR)	25.2 (5.5)	25.6 (4.1)	1.02 (0.94‐1.10)
Smoking status, n (%)			
Yes	31 (14.0)	4 (13.3)	1
No	190 (86.0)	26 (86.7)	1.06 (0.35‐3.25)
Aspirin intolerance, n (%)			
Yes	10 (4.4)	1 (3.3)	1
No	215 (95.6)	29 (96.7)	1.35 (0.17‐10.93)
Environmental allergy, n (%)			
Yes	70 (31.1)	13 (43.3)	1.69 (0.78‐3.68)
No	155 (68.9)	17 (56.7)	1
Bronchial asthma, n (%)			
Yes	60 (26.4)	11 (36.7)	1.61 (0.73‐3.58)
No	167 (73.6)	19 (63.3)	1
Preoperative peroral corticosteroids, n (%)			
Yes	18 (7.9)	3 (10.0)	1.30 (0.36‐4.69)
No	210 (92.1)	27 (90.0)	1
Elixhauser Comorbidity Index, median (IQR)	0 (3)	0 (1)	1.0 (0.87‐1.13)
ASA classification, n (%)			
1‐2	165 (83.8)	18 (85.7)	1.16 (0.32‐4.18)
3‐4	32 (16.2)	3 (14.3)	1
Lund‐MacKay score, median (IQR)	8.5 (7)	9 (6)	1.04 (0.95‐1.15)
Zinreich score, median (IQR)	2 (4)	2.5 (4)	1.12 (0.96‐1.31)
Right frontal recess status, n (%)			
Open	83 (39.0)	12 (44.4)	1.25 (0.56‐2.81)
Closed	130 (61.0)	15 (55.6)	1
Left frontal recess status, n (%)			
Open	90 (42.1)	11 (40.7)	1
Closed	124 (57.9)	16 (59.3)	1.06 (0.47‐2.38)
Previous sinonasal surgeries, n (%)			
Yes	115 (50.4)	20 (66.7)	1.97 (0.88‐4.38)
No	113 (49.6)	10 (33.3)	1
Indication			
Infections	102 (47.9)	16 (55.2)	3.14 (1.01‐9.75)^a^
Pain	80 (37.6)	4 (13.8)	1^a^
Pressure adjusting problems	27 (12.7)	7 (24.1)	
Enlarging a previously dilated frontal canal	4 (1.9)	2 (6.9)	
Form of anesthesia, n (%)			
General	188 (82.5)	25 (83.3)	1.06 (0.38‐2.95)
Local	40 (17.5)	5 (16.7)	1
Deviced used, n (%)			
Acclarent	24 (10.5)	1 (3.3)	1
Entellus	47 (20.6)	4 (13.3)	2.04 (0.22‐19.30)
Unknown	157 (68.8)	25 (83.3)	

Abbreviations: BMI, bodymass index; CI, confidence interval; IQR, interquartile range; OR, odds ratio.

^a^ Analysis performed between infections and pain due to small group sizes for the other indications.

The primary reasons for failure in technically failed cases were problems inserting the balloon (n = 28), repeatedly broken balloon (n = 1), or poor visibility due to bleeding (n = 1). Causes for problems with balloon insertion included sclerosis, inability to find the frontal recess, and a narrow frontal recess.

Compared to patients with pain as indication, patients with infection as indication experienced technical failures significantly more often (odds ratio [OR] = 3.14 95% confidence interval [CI] 1.01‐9.75). The technically failed and successful balloon sinuplasty groups were similar regarding age, sex, bodymass index (BMI), smoking status, aspirin intolerance, environmental allergy, bronchial asthma, preoperative peroral corticosteroids, previous sinonasal surgeries, Elixhauser Comorbidity Index, Lund‐MacKay score, Zinreich score, status of the frontal recesses, and device used.

### Preoperative Factors

The indication for the initial operation was infection or inflammation (including acute, recurrent acute, or chronic sinusitis) in 120 operations (49.2%), pain (84; 34.4%), problems adjusting pressure (34; 13.9%), and enlarging a previously dilated frontal recess (6; 2.5%). In the last group, 1 patient had undergone surgery for papilloma (2 postoperative balloon sinuplasties), 1 had a drained mucocele (1 postoperative sinuplasty), and 1 had a chronic frontal sinusitis due to an immune deficiency (3 postoperative sinuplasties). The indication was unrecorded in 14 patients (5.4%). Two had been diagnosed with nasal polyposis preoperatively.

### Imaging Studies

The median (IQR) preoperative Lund‐MacKay score was 9 (7); the median (IQR) preoperative Zinreich score was 2 (4). The status of the frontal recess in preoperative CT scans was open on both sides in 59 patients (23.0%), open on 1 side in 78 (30.2%), and closed on both sides in 104 (40.5%). In 17 cases (6.6%), the preoperative status was unknown. Of those with a bilaterally closed frontal recess, 77 patients (74.0%) had a bilateral sinuplasty, whereas 42 (53.8%) with unilaterally closed frontal recess and 34 (57.6%) with bilaterally open frontal recess had bilateral sinuplasty.

### Intraoperative Factors

Most of the patients (n = 169; 65.5%) underwent 1 or more simultaneous sinonasal procedures, that is, a hybrid procedure. These included surgery to other sinuses through conventional methods (n = 140), including opening of agger nasi or supraorbital cells (n = 17), balloon dilation of maxillary or sphenoidal sinuses (n = 19), minitrephination (n = 28), and polypectomy (n = 28). Seventy patients underwent a balloon only procedure. One patient had a technically successful balloon sinuplasty in their left frontal recess, whereas the right 1 failed, and was operated in a conventional manner.

### Complications

Within the first 30 days after surgery, there were 27 (10.5%) complications, of which 23 occurred after hybrid surgery, and 3 after a balloon only procedure. Two (7.4%) were postoperative bleedings that required treatment, and 25 (92.6%) were postoperative infections, of which 16 had a preoperative infection. Antibiotics were started preoperatively in cases with preoperative infection, and intraoperatively if pus was found during operation. In case of intraoperative pus findings, bacterial cultures were taken and the antibiotics were targeted thereafter. The length of antibiotic treatment was 7 days, in accordance with Finnish guidelines on antibiotic treatment for acute rhinosinusitis. No antibiotics were given if there were no signs of infection. There were no intraoperative complications, orbital fractures, skull base injuries, or intracranial injuries.

### Reoperations

Of all patients undergoing frontal sinus balloon sinuplasty, 38 patients (15.7%) underwent a reoperation, including the 7 patients undergoing repeat balloon sinuplasties. In the balloon only group, the reoperation rate was 26.8%, whereas it was 10.7% in the hybrid group (OR = 3.07, 95% CI 1.50‐6.28). The 7 patients undergoing repeat balloon sinuplasties underwent altogether 17 reoperations, of which 15 were balloon only procedures, and 2 were hybrid procedures. The rest of the reoperations were performed as conventional ESS. The median time interval (IQR) between initial surgery and reoperation was 5 months (13). The indications for reoperation were pain (15 patients; 39.5%), postoperatively constricted frontal recess (10; 26.3%), infections (8; 21.1%), problems adjusting pressure (4; 10.5%), and discharge (1; 2.6%). The indication for the reoperation was the same as for the primary operation in 25 patients (65.8%).

Supplemental Table S1, available online shows factors predicting reoperation. Patients with previous sinonasal surgery had significantly more reoperations than patients who underwent their first sinonasal operation (OR = 3.03, 95% CI 1.40‐6.56), as did patients who underwent their balloon sinuplasty under local anesthesia compared to general anesthesia (OR = 4.77, 95% CI 2.23‐10.20). Patients who reported benefit from the balloon sinuplasty had a lower reoperation rate (OR = 0.11, 95% CI 0.04‐0.33). The group with other simultaneous sinonasal surgeries had a lower reoperation rate than balloon surgery alone (OR = 0.33, 95% CI 0.16‐0.67). Other simultaneous sinonasal surgeries included both surgery to other than frontal sinuses, minitrephination, as well as opening agger nasi and supraorbital cells with conventional technique. Acclarent device (n = 25, 9.7%) and lack of postoperative packing predicted a higher reoperation rate (OR = 11.29, 95% CI 2.18‐58.53 and OR = 3.53, 95% CI 1.62‐7.67, respectively).

Age, sex, BMI, smoking status, aspirin intolerance, environmental allergy, bronchial asthma, Elixhauser Comorbidity Index, imaging findings, ASA classification, preoperative use of peroral corticosteroids, or intraoperative sinus findings did not affect the reoperation rate.

Among those answering the questionnaire, a higher reoperation rate was noted in patients who were recommended nasal corticosteroids postoperatively (OR = 6.91, 95% CI 0.87‐54.8), those with forehead pain during infections (OR = 4.89, 95% CI 1.08‐22.02), and those who had no reason for their pain (OR = 2.63, 95% CI 0.99‐6.99).

### Questionnaire and Factors Predicting Outcome

Of the 242 patients who had a successful balloon sinuplasty, 156 (64.5%) returned the follow‐up questionnaire (Table 1). The median time interval (IQR) between the surgery and questionnaire was 4.5 years (3 years).

The group answering the questionnaire had similar characteristics as the nonanswering 1, except for the number of patients having other surgeries simultaneously (n = 119, 76.8% in the answering group; n = 50, 58.8% in the nonanswering group, *p* = .005).

Subjectively, 138 patients (88.5%) benefitted from frontal balloon sinuplasty. In the balloon only group, patient satisfaction rate was 91.7%, whereas it was 87.4% in the hybrid group. Subjective patient satisfaction was associated with a low SNOT‐22 score (OR = 0.97, 95% CI 0.95‐0.99).

Patient satisfaction was higher in patients who did not require antibiotics for sinusitis (OR = 3.87, 95% CI 1.37‐10.97), used nasal corticosteroid treatment during the previous 12 months (OR = 8.26, 95% CI 1.06‐64.24), or were currently asymptomatic (Supplemental Table S2, available online). Patient characteristics, CT scores, indication for sinus surgery, intraoperative factors, or any other measures studied in the questionnaire did not affect patient satisfaction.

## Discussion

The technical success rate of frontal balloon sinuplasty in our study was high (93.6%), and most patients (88.5%) reported a beneficial long‐term outcome. Regular postoperative use of nasal corticosteroids was associated with higher patient satisfaction. However, we could not identify any preoperative predictors for patient satisfaction. Although these patients could not terminate the use of nasal corticosteroids postoperatively, it is possible that successful balloon sinuplasty enabled the penetration of medical treatment, relieving the disease.

The reoperation rate was 15.7%. We found hybrid procedures and general anesthesia to be protective factors for reoperation, whereas previous sinonasal surgeries was associated with a higher reoperation rate. Interestingly, the reoperation rate was also higher among those using regular postoperative nasal corticosteroids. The reoperation rate is somewhat higher than previously reported for balloon‐only procedures but similar to that of hybrid procedures. In 2018, Chaaban et al performed a large retrospective study on 16,040 patients undergoing sinus surgery through balloon sinuplasty, conventional ESS, or a hybrid procedure. The reoperation rate after 6 months was 7.9% in the balloon sinuplasty group, while the rate in the hybrid group was 15.2% and 16.9% in the ESS group.^24^ In other studies with varying follow‐up times, the revision rate for balloon only procedures has been reported as 3.0% to 7.4% and 0% to 6.3% for hybrid procedures.^3^, ^25^, ^26^, ^27^ Our higher revision rate may be explained by the longer follow‐up time than previous studies.

In contrast to the Chaaban et al^24^ study, we found that concurrent sinus surgeries adjunct to the frontal balloon sinuplasty lead to a lower reoperation rate, suggesting that whenever indicated, a hybrid approach would decrease the risk of reoperation, as these conventional methods also participate in improving sinus drainage and medication delivery. In the hybrid cases of our study, conventional methods were mainly used in reaching sinuses other than frontal sinuses. Hence, balloon dilation might be useful in frontal sinus surgery, whereas conventional methods might be preferable in the other sinuses.

The reoperation rate after balloon sinuplasty under general versus local anesthesia has not been studied before. It is possible that general anesthesia allows the surgeon to perform a more extensive surgery, thus offering a better relief of the disease.

In our study, patients with previous sinonasal surgeries had a higher reoperation rate, as did patients who had undergone new sinonasal surgeries outside of our clinic after the balloon sinuplasty. Hence, a traditional approach seems to be preferable over balloon sinuplasty in revision cases. A history of sinonasal surgery could indicate a more severe illness, in which sole balloon sinuplasty might be insufficient. Postoperative packing was also connected with a lower reoperation rate, presumably because packing was more often used in hybrid surgeries. Unlike in Chaaban et al's study, comorbidities did not predict complications or reoperations in our study.^24^ We found no association between preoperative imaging results and the reoperation rate. In 2018, Piccirillo et al published a clinical consensus statement on balloon dilation of the sinuses, stating that CT imaging is required preoperatively and that balloon sinuplasty is not indicated in patients without sinonasal disease findings on CT scans.^9^ Preoperative imaging also helps preoperative planning, and cases where the clinician evaluated that balloon sinuplasty would be impossible were, naturally, not included in our study.

Our technical failure rate (6.4%) is in accordance with that of previous reports (6%‐19%).^12^, ^13^, ^28^, ^29^ We found an association between infection as indication for surgery and technical failure. It is possible, that the mucosal oedema during infection makes balloon insertion into the frontal recess harder. In 2011, Heimgartner et al showed an association between preoperative CT scans and technical failure.^12^ We agree with Heimgartner et al that the learning curve can affect technical failure, as the most prevalent reason for technical failure in our material was “failure to identify the frontal recess.” The learning curve could also explain Acclarent's higher failure rate, as it was the first device used in our hospital. Although we did not find preoperative CT scans predictive of failure, anatomy likely also plays a role in the failure of inserting the balloon catheter.

Our material included 59 patients undergoing frontal sinus balloon sinuplasty despite of having bilaterally open frontal recesses in preoperative CT scans, which might seem controversial. As the indication for frontal sinus surgery is mostly symptom‐based, we do not consider a radiologically open frontal recess as a contradiction for sinuplasty. The patient may carry a heavy history of recurrent infections or pressure‐adjusting pain episodes, and yet present an open frontal recess on CT scans taken between these symptom episodes.

This study's strengths are its long follow‐up, large sample size, and the questionnaire's good response rate. The study's partly retrospective nature can be seen as a possible weakness. The group responding to the questionnaire might have been affected by selection bias, and additionally, due to the long duration between a procedure and answering the questionnaire, recall bias is possible. Selection bias is also possible between the groups undergoing a hybrid procedure versus a balloon only procedure, as a more extensive procedure might indicate a more severe disease. The inclusion of patients with nasal polyposis or aspirin‐exacerbated respiratory disease might affect the results of the study, however, they were not excluded from the material as it included all balloon sinuplasties performed at our clinic during the predetermined time period. Since the study was performed at a public hospital, the fees were covered by the patient's municipalities, and the price of the used instrumentation did not affect the surgeon's choice of operation technique. Due to this, the treatment options discussed in this study might not be comparable to that of countries with other payment systems.

## Conclusion

In this study, combining retrospective data and a patient questionnaire on frontal sinus balloon dilation, we found a technical success rate of 93.6% and a patient satisfaction rate of 88.5%. Regular postoperative use of nasal corticosteroids was associated with higher patient satisfaction. Previous sinonasal surgeries presented as a predicting factor for a higher reoperation rate. Thus, balloon sinuplasty seems insufficient in revision cases. A hybrid approach as well as general anesthesia appear to be protective factors for reoperations.

## Author Contributions

**Sara Sainio**, study conception and design, data collection, statistical analysis, writing and reviewing; **Karin Blomgren**, study conception and design, data collection, writing and reviewing; **Anni Koskinen**, study conception and design, data collection, writing and reviewing; **Marie Lundberg**, study conception and design, data collection, writing and reviewing.

## Disclosures

### Competing interests

None.

### Sponsorships

None.

### Funding source

None.

## Supporting information

Supporting information.

Supporting information.
