# Supplementary material for: Frontal Sinus Balloon Sinuplasty—Patient Satisfaction and Factors Predicting Reoperation
Source: OTO Open. 2023 Mar 22;7(1):e23. doi: 10.1002/oto2.23 (PMC10046702; doi:10.1002/oto2.23)
Supplement: Supplementary file 1 — Supporting information. [file OTO2-7-e23-s002.docx]

**Supplemental Table 1:** Factors predicting reoperation in patients who underwent frontal sinus balloon sinuplasty at Helsinki University Hospital (HUS) from 2008 to 2019.

|  |  | **No reoperation, n (%)** | **Reoperation at HUS, n (%)** | **OR (95% CI)** | **P-value** |
| --- | --- | --- | --- | --- | --- |
| Sex | Women  Men | 105 (51.5)  99 (48.5) | 17 (44.7)  21 (55.3) | 1  1.31 (0.65–2.63) | 0.45 |
| Age, mean (95% CI) |  | 44.2 (42.2–46.2) | 47.7 (42.9–52.5) | 1.02 (0.99–1.04) | 0.17 |
| BMI, median (IQR) |  | 25.5 (5.3) | 24.8 (5.9) | 1.022 (0.95–1.10) | 0.78 |
| Smoking status | Yes  No | 31 (15.7)  167 (84.3) | 2 (5.4)  35 (94.6) | 1  3.25 (0.74–14.21) | 0.10 |
| Aspirin intolerance | No  Yes | 192 (95.5)  9 (4.5) | 36 (94.7)  2 (5.3) | 1  1.19 (0.25–5.71) | 0.83 |
| Environmental allergy | No  Yes | 138 (68.7)  63 (31.3) | 24 (63.2)  14 (36.8) | 1  1.28 (0.62–2.63) | 0.51 |
| Bronchial asthma | No  Yes | 150 (73.9)  53 (26.1) | 25 (65.8)  13 (34.2) | 1  1.47 (0.70–3.08) | 0.30 |
| Preoperative peroral corticosteroids | No  Yes | 188 (92.2)  16 (7.8) | 35 (92.1)  3 (7.9) | 1  1.01 (0.30–3.64) | 0.99 |
| Elixhauser comorbidity index, median (IQR) |  | 0 (3) | 0 (5) | 1.0 (0.88–1.13) | 0.71 |
| ASA classification | 1–2  3–4 | 147 (83.5)  29 (16.5) | 27 (87.1)  4 (12.9) | 1.33 (0.43–4.09)  1 | 0.62 |
| Lund-MacKay score, median (IQR) |  | 8 (8) | 9.5 (6) | 1.05 (0.95–1.15) | 0.33 |
| Zinreich score, median (IQR) |  | 2 (4) | 3.5 (3) | 1.09 (0.93–1.28) | 0.19 |
| Right frontal recess status | Open  Closed | 79 (39.7)  120 (60.3) | 9 (33.3)  18 (66.7) | 1  1.32 (0.56–3.08) | 0.52 |
| Left frontal recess status | Open  Closed | 84 (42.0)  116 (58.0) | 11 (40.7)  16 (59.3) | 1  1.05 (0.46–2.39) | 0.90 |
| Previous sinonasal operations | No  Yes | 106 (52.0)  98 (48.0) | 10 (26.3)  28 (73.7) | **1**  **3.03 (1.40–6.56)** | **0.004** |
| Indication | Infections  Pain  Pressure adjusting problems  Enlarging a previously dilated frontal canal | 95 (48.2)  74 (37.6)  27 (13.7)  1 (0.5) | 15 (50.0)  8 (26.7)  4 (13.3)  3 (10.0) | 1.46 (0.59–3.63)*  1 | 0.41* |
| Form of anesthesia | General  Local | 177 (86.8)  27 (13.2) | 22 (57.9)  16 (42.1) | **1**  **4.77 (2.23–10.20)** | **<0.001** |
| Used dilation device | Acclarent  Entellus  Unknown | 17 (8.3)  48 (23.5)  139 (68.1) | 8 (21.1)  2 (5.3)  28 (73.7) | **11.29 (2.18–58.53)**  **1** | **<0.001** |
| Other simultaneous sinonasal operations | No  Yes | 52 (25.6)  151 (74.4) | 19 (51.4)  18 (48.6) | **3.07 (1.50–6.28)**  **1** | **0.002** |
| Frontal sinus findings | Pus/polyp  Clean | 31 (15.2)  173 (84.8) | 10 (26.3)  28 (73.7) | 2.06 (0.91–4.67)  1 | 0.09 |
| Packing | No  Yes | 89 (43.6)  115 (56.4) | 28 (73.7)  10 (26.3) | **3.53 (1.62–7.67)**  **1** | **<0.001** |
| **Questionnaire:** |  |  |  |  |  |
| Have you used antibiotics for sinusitis during the last 12 months? | Yes  No | 50 (36.5)  87 (63.5) | 9 (47.4)  10 (52.6) | 1.57 (0.60–4.11)  1 | 0.36 |
| Have you used nasal corticosteroids during the last 12 months? | Yes  No | 90 (66.2)  46 (33.8) | 17 (89.5)  2 (10.5) | 4.34 (0.96–19.62)  1 | 0.06 |
| Was regular nasal corticosteroid use recommended postoperatively? | No  Yes | 29 (31.5)  63 (68.5) | 1 (6.3)  15 (93.8) | 1  6.91 (0.87–54.8) | **0.04** |
| Do you regularly use any other local nasal treatment(s)? | Yes  No | 63 (46.0)  74 (54.0) | 12 (36.8)  7 (63.2) | 1  2.01 (0.75–5.42) | 0.16 |
| Do you smoke daily? | Yes  No | 12 (8.8)  125 (91.2) | 0  19 (100) | **1**  **1.15 (1.08–1.23)** | 0.18 |
| Have you had any other sinonasal surgeries performed outside Helsinki University Hospital after your balloon sinuplasty? | Yes  No | 1 (0.7)  136 (99.3) | 2 (10.5)  17 (89.5) | **16 (1.38–185.94)**  **1** | **0.004** |
| Problems adjusting sinus pressure? | Yes  No | 53 (38.7)  84 (61.3) | 10 (52.6)  9 (47.4) | 1.76 (0.67–4.62)  1 | 0.25 |
| Pain during airway infections? | No  Yes | 50 (36.5)  87 (63.5) | 2 (10.5)  17 (89.5) | **1**  **4.89 (1.08–22.02)** | **0.02** |
| Pain without any specific reason? | No  Yes | 90 (65.7)  47 (34.3) | 8 (42.1)  11 (57.9) | 1  2.63 (0.99–6.99) | **0.046** |
| Do you think you benefitted from the operation? | Yes  No | 127 (92.7)  10 (7.3) | 11 (57.9)  8 (42.1) | **1**  **9.24 (3.03–28.17)** | **<0.001** |
| SNOT-22, median (IQR) |  | 22 (25) | 22 (31) | 1.00 (0.98–1.03) | 0.79 |

* Analysis performed between infections and pain due to small group sizes for the other indications.
