# Supplementary material for: Frontal Sinus Balloon Sinuplasty—Patient Satisfaction and Factors Predicting Reoperation
Source: OTO Open. 2023 Mar 22;7(1):e23. doi: 10.1002/oto2.23 (PMC10046702; doi:10.1002/oto2.23)
Supplement: Supplementary file 2 — Supporting information. [file OTO2-7-e23-s001.docx]

**Supplemental Table 2:** Factors predicting patient satisfaction in patients who underwent frontal sinus balloon sinuplasty at Helsinki University Hospital from 2008 to 2019.

|  |  | **Did not benefit, n (%)** | **Benefitted, n (%)** | **OR (95% CI)** | **P-value** |
| --- | --- | --- | --- | --- | --- |
| Sex | Women  Men | 9 (50.0)  9 (50.0) | 76 (55.1)  62 (44.9) | 1.23 (0.46–3.28)  1 | 0.68 |
| Age, mean (95% CI) |  | 40.2 (33.2–47.2) | 47.0 (44.7–49.4) | 1.04 (1.0–1.08) | 0.05 |
| BMI, median (IQR) |  | 24.0 (5.6) | 25.5 (5.7) | 1.08 (0.96–1.22) | 0.12 |
| Smoking status | No  Yes | 16 (94.1)  1 (5.9) | 120 (88.2)  16 (11.8) | 1  2.13 (0.27–17.19) | 0.47 |
| Aspirin intolerance | No  Yes | 16 (88.9)  2 (11.1) | 130 (94.9)  7 (5.1) | 2.32 (0.44–12.15)  1 | 0.31 |
| Environmental allergy | No  Yes | 13 (72.2)  5 (27.8) | 93 (67.9)  44 (32.1) | 1  1.23 (0.41–3.67) | 0.71 |
| Bronchial asthma | No  Yes | 14 (77.8)  4 (22.2) | 98 (71.0)  40 (29.0) | 1  1.43 (0.44–4.61) | 0.55 |
| Preoperative peroral corticosteroids | No  Yes | 16 (88.9)  2 (11.1) | 127 (92.0)  11 (8.0) | 1.44 (0.29–7.10)  1 | 0.65 |
| Elixhauser comorbidity index, median (IQR) |  | 0 (3) | 0 (3) | 0.93 (0.77–1.13) | 0.44 |
| ASA classification | 1–2  3–4 | 13 (81.3)  3 (18.8) | 104 (86.7)  16 (13.3) | 1.5 (0.39–5.85)  1 | 0.56 |
| Lund-MacKay score, median (IQR) |  | 8 (8) | 8 (6) | 1.03 (0.92–1.15) | 0.73 |
| Zinreich score, median (IQR) |  | 2 (3) | 2 (4) | 1. (0.81–1.24) | 0.85 |
| Right frontal recess status | Open  Closed | 7 (38.9)  11 (61.1) | 55 (41.0)  79 (59.0) | 1.09 (0.40–3.00)  1 | 0.86 |
| Left frontal recess status | Open  Closed | 9 (50.0)  9 (50.0) | 56 (41.5)  79 (58.5) | 1  1.41 (0.53–3.78) | 0.49 |
| Previous sinonasal operations | No  Yes | 10 (55.6)  8 (44.4) | 60 (43.5)  78 (56.5) | 1  1.63 (0.61–4.37) | 0.33 |
| Indication | Infections  Pain  Pressure adjusting problems Enlarging a previously dilated frontal canal | 8 (44.4)  8 (44.4)  2 (11.1)  0 | 63 (47.4)  52 (39.1)  17 (12.8)  1 (0.8) | 1.21 (0.43–3.45)*  1 | 0.72* |
| Form of anesthesia | General  Local | 15 (83.3)  3 (16.7) | 121 (87.7)  17 (12.3) | 1.42 (0.73–5.43)  1 | 0.60 |
| Used dilatation device | Acclarent  Entellus  Unknown | 3 (16.7)  3 (16.7)  12 (66.7) | 14 (10.1)  33 (23.9)  91 (65.9) | 1  2.36 (0.42–13.14) | 0.32 |
| Other simultaneous sinonasal operations | No  Yes | 3 (16.7)  15 (83.3) | 33 (24.1)  104 (75.9) | 1.59 (0.43–5.82)  1 | 0.77 |
| Frontal sinus findings | Pus/polyps  Clean | 4 (22.2)  14 (77.8) | 20 (14.5)  118 (85.5) | 1  1.69 (0.50–6.64) | 0.39 |
| Packing | No  Yes | 9 (50.0)  9 (50.0) | 60 (43.5)  78 (56.5) | 1  1.3 (0.49–3.48) | 0.60 |
| **Questionnaire:** |  |  |  |  |  |
| Have you used antibiotics for sinusitis during the last 12 months? | Yes  No | 12 (66.7)  6 (33.3) | 47 (34.1)  91 (65.9) | **1**  **3.87 (1.37–10.97)** | **0.007** |
| Have you used nasal corticosteroids during the last 12 months? | No  Yes | 1 (5.9)  16 (94.1) | 47 (34.1)  91 (65.9) | **8.26 (1.06–64.24)**  **1** | **0.02** |
| Was regular nasal corticosteroid use recommended postoperatively? | No  Yes | 3 (30.0)  7 (70.0) | 27 (27.6)  71 (72.4) | 1  1.13 (0.27–4.68) | 0.87 |
| Do you regularly use any other local nasal treatment(s)? | No  Yes | 8 (44.4)  10 (55.6) | 73 (52.9)  65 (47.1) | 1.40 (0.52–3.77)  1 | 0.50 |
| Do you smoke daily? | Yes  No | 1 (5.6)  17 (94.4) | 11 (8.0)  127 (92.0) | 1.47 (0.18–12.13)  1 | 0.72 |
| Have you had any other sinonasal surgeries performed outside Helsinki University Hospital after your balloon sinuplasty? | Yes  No | 2 (11.1)  16 (88.9) | 1 (0.7)  137 (99.3) | **1**  **17.13 (1.47–199.58)** | **0.003** |
| Problems adjusting sinus pressure? | No  Yes | 6 (33.3)  12 (66.7) | 87 (63.0)  51 (37.0) | **3.41 (1.21–9.64)**  **1** | **0.02** |
| Pain during airway infections? | No  Yes | 1 (5.6)  17 (94.4) | 51 (37.0)  87 (63.0) | **9.97 (1.29–77.12)**  **1** | **0.008** |
| Pain without any specific reason? | No  Yes | 7 (38.9)  11 (61.1) | 91 (65.9)  47 (34.1) | **3.04 (1.11–8.36)**  **1** | **0.03** |
| SNOT-22 score, median (IQR) |  | 29.5 (48) | 21 (26) | **0.97 (0.95–0.99)** | **0.03** |

* Analysis performed between infections and pain due to small group sizes for the other indications.
